# Consequences of hypertension and chronic obstructive pulmonary disease, healthcare-seeking behaviors of patients, and responses of the health system: a population-based cross-sectional study in Bangladesh

**DOI:** 10.1186/1471-2458-14-547

**Published:** 2014-06-03

**Authors:** Md Jasim Uddin, Nurul Alam, Haribondhu Sarma, Muhammad Ashique Haider Chowdhury, Dewan S Alam, Louis Niessen

**Affiliations:** 1 International Centre for Diarrhoeal Disease Research Bangladesh (icddr,b), Mohakhali, Dhaka 1212, Bangladesh; 2 Centre for Applied Health Research and Delivery, Suite 1966–206, Liverpool School of Tropical Medicine/Medical School, University of Warwick Pembroke Place, Liverpool L3 5QA, UK

**Keywords:** Non-communicable diseases, Hypertension, Chronic obstructive, Pulmonary disease, Comorbidity, Health-care behaviour, Health system, Bangladesh

## Abstract

**Background:**

Non-communicable diseases are a threat to human health and economic development of low-income countries. Hypertension (HT) and chronic obstructive pulmonary disease (COPD) are two major causes of deaths, worldwide. This study assesses the health status, health-care seeking, and health provider responses among patients with these conditions.

**Methods:**

The study carried out population-based cross-sectional survey in a rural and an urban surveillance area in Bangladesh. It interviewed all patients identified with HT and COPD at home using a structured questionnaire on the health consequences, healthcare-seeking behaviours, and coping strategies. Qualitative techniques identified key factors relating to the behaviours of patients and providers.

**Results:**

COPD and HT correlate with lower activities of daily living (ADL) scores. The odds ratio (OR) for ADL scores in the combied conditions are high (OR: 3.04, p < 0.05) as compared to hypertension. Financial crises occur significantly more frequently among COPD patients in the urban site as compared to those in rural ares (12.5% vs. 2.4%, p < 0.01). Self-treatment at the onset is common. Seeking care from trained providers is higher in urban settings and is higher for HT. Referral for both COPD and hypertension was inadequate until the disease severity increased.

**Conclusions:**

COPD and HT significantly are associated with lower ADL scores and financial problems. Public-sector primary healthcare facilities should be better organised to address both conditions with the aim to reduce household poverty.

## Background

Non-communicable diseases (NCDs) are a global threat to human health and the development and economy in low-income countries. The 2010 global status report of the World Health Organization (WHO) on NCDs, shows that NCDs, particularly cardiovascular diseases, cancers, diabetes, and lung diseases, have emerged as leading causes of mortality in the world [1,2]. NCD prevalence is steadily increasing, including among working age populations [3]. Recent studies indicate that COPD and hypertension (HT) are among the most common causes of mortality [4,5].

COPD is a progressively disabling disease of the lower respiratory tract. In addition to the risk of dying, a major concern is the occurrence of comorbidities. Abot 50% of COPD patients have at least one other chronic disease and among them hypertension is the most frequent [6,7]. HT is the leading risk factors and contributes to chronic cardiovascular disease occurrence, ischemic heart diseases and stroke. COPD severity is reported as associated with increased risk of hypertension [6].

In a review of 23 developing countries Bangladesh shows the 9th highest rate of age-standardized mortality due to NCDs, primarily including cardiovascular diseases (CVDs) and diabetes [7]. A recent hospital study across Bangladesh shows that about one-third of admissions of patients aged over 30 years are due to the major NCDs [7].

As a leading cause of mortality and morbidity among those over 40 years of age, COPD shows one of the most alarming NCDs increases in Bangladesh. COPD prevalence is 13% among men and women aged 40 years and above [6]. The two most important causes of COPD are smoking and indoor air pollution [8-10]. COPD accounted for 559/100,000 disability-adjusted life-years, ranking Bangladesh in top five countries with the highest prevalence [11]. The Bangladesh Demographic and Health Survey 2011 reports that HT prevalence is 32% among adults aged ≥35 years [12]. A more recent study reports that HT prevalence is 12.4% in rural areas and 16.1% in urban areas [13]. The National NCD Risk Factor Survey of 2010 shows that about 11% of women and 8% of men were aware of these conditions but did not seek any treatment [14]. About 79% of HT patients does not take regular treatment for their sickness [15].

It is clear that COPD and HT are important public-health problems in Bangladesh. The demographic transition and changes in lifestyle, along with increased rates of urbanization, are major factors in the rise of these conditios. As the disease burden shifts towards higher ages, the health systems of the country face growing demands of both rich and poor people [16]. The present national essential services package does not include prevention or the treatment of chronic diseases. The annual ‘Reality Check’ study of the ultra poor observes an increasing demand of care for both condition and highlights the need for information on the societal consequences of COPD and HT, healthcare-seeking behaviour, and services availability and their community responsiveness [17].

This article describes the consequences of HT and COPD on daily functioning of patients, healthcare-seeking behaviour, and provider responses.

## Methods

This cross-sectional study was conducted during 2012–2013. The study used a mixed method approach i.e. both quantitative and qualitative techniques were applied. The qualitative data were collected after completion of a patient survey for complementing survey data. Qualitative research among the sampled households explored key topics raised after initial analyses of the survey data. The survey questionnaire examined the consequences of COPD and HT. Descriptive qualitative techniques examined the consequences of COPD and HT, healthcare seeking behaviours, and the responses of the health systems. The qualitative data lead to a more indepth understanding of the process and mechanisms in the use of health care and bring in the topic raised by those health care providers who are consulted most often.

### Definitions

*Hypertension* was defined as systolic blood pressure of ≥140 mm hg or diastolic blood pressure of ≥90 mm hg or being on antihypertensive medication without showing systolic and diastolic measures of above normal [Alam S 2010, an unpublished research protocol on HT].

*COPD* is a lung disease characterized by chronic obstruction of lung airflow that interferes with normal breathing and is not fully reversible [Alam S 2010, an unpublished research protocol on COPD].

*ADL* scores are describing the level of those activities of daily living that indicate the functional status of a person, referring to daily care activities within the place of residence, in the outdoor environments, or both.

*Financial crisis*: a situation in which the income of a family experiences suddenly reduces as a consequence of an acute or chonic disease.

### Health service provision in study sites

In the study sites, the distribution of public healthcare services and facilities follows the same pattern of administrative tiers ranging from national (mostly capital-based in Dhaka), regional (in divisions), district, sub-district, to community level provisions. Secondary and tertiary care in both urban and rural sites is the sole responsibility of the Minsitry of Health and Family Welfare (MoHFW).

At the rural sites, the Upazila (sub-district) Health Complex (UHC) is a fixed service-delivery point designed to provide first-level referral services to the people. The UHC is meant for curative, preventive, promotive and rehabilative health services using the hospital facility and by field-level workers. In each UHC, there are posts for nine doctors, including one Upazila Health and Family Planning Officer (UHFPO). UHFPO, the Chief Health Officer of the upazila, is also the head of the UHC. Other doctors in the UHC include junior consultants-4, residential medical officer-1, assistant surgeon-2, and dental surgeon-1. The UHC provides outpatient, inpatient, and emergency services, limited diagnostic and imarging services, emergency obstetric care, maternal healthcare, and dental care.

Below Upazila level there are three types of static health facilities at the union level (8–10 unions in each upazila). These are Union Subcentres, Union Health and Family Welfare Centres (UHFWCs) and Community Clinics (CCs). Service providers at the union-level facilities include Medical Officers/Assistant Surgeons, Medical Assistant (MA), Pharmacist, and member of lower service staff (MLSS).

The main health workforce at community level is the domiciliary staff called Health Assistants (HAs). They are placed in community level. They make home-visits to provide primary health care (PHC) services and collect routine health data. The HAs routinely organize outreach centres for PHC services.

In urban sites, the Ministry of Local Government, Rural Development and Cooperative (MoLGRDC) is responsible for health matters, which are executed by the city corporation/municipality authorities. City corporation/municipal authorities are supported by various government and non-government organizations and private-sector agencies that also provide services in urban sites. The government sector, including municipal health department, and NGOs concentrate on preventive and promotive healthcare while the private sector concentrates mainly on curative healthcare.

The traditional medicine systems practiced in Bangladesh include the Unani and Ayurvedic which have a joint governing board, although each has its own network of teaching colleges. The expansive informal sector includes traditional birth attendants, drug vendors, and village doctors.

### Study sites and subjects

The study was conducted in one rural (Matlab) and one urban (Kamalapur) surveillance sites of the International Centre for Diarrhoeal Disease Research, Bangladesh (icddr,b) and included (a) patients with hypertension, (b) patients with COPD, or (c) patients with both conditions living in the study sites, and (d) healthcare providers working within the study areas.

### Sampling

The sampled households (N = 989) were identified and sampled from an existing list of households identified earlier as having at least one patient with COPD or hypertension, living in Matlab (n = 483) or in Kamalapur (n = 506). The list was the result from previously conducted epidemiological studies [Alam S 2010, unpublished research protocols on HT and COPD]. The present study included all patients identified through the screening process of the studies [Alam S 2010, unpublished research protocols on HT and COPD]. The earlier studies were population-based on the prevalence and determinants of hypertension and COPD [Alam S 2010, an unpublished research protocol on HT] and a follow-up study to assess of rate of decline in pulmonary function in the diagnosed COPD cases [Alam S 2010, an unpublished research protocol on COPD].

In total, 3758 people were interviewed in each study site to measure the prevalence of hypertension. Here, age-stratified lists of all the subjects were obtained from the database of the Health and Demographic Surveilance system of icddr,b. For each age stratum, the required number of individuals was randomly selected. The availability of subjects was verified through a door-to-door visit. The study team contacted each selected individual to explain the study objectives, invited them for participation in the study and detected patients following standered procedure.

#### Study inclusion criteria

The study included all adult males and non-pregnant females, aged ≥20 years, who did not suffer from any heart attack or stroke, who had no history of heart or kidney failure, not suffering from any acute illness during recruitment, and who were able to provide valid consent for participation, and were willing to participate in the study.

For COPD, the individuals were randomly selected by Alam’s study [Alam S 2010, an unpublished research protocol on COPD] for examination with spirometry for dictation. In total, 3758 individuals were randomly tested through spirometry in each site to identify COPD patients. Here, the included age group were the ≥40-year-old individuals.

### Data collection

All the patients of hypertension, COPD, and comorbidity clinically diagnosed during the epidemiological study [Alam S 2010, unpublished research protocols on HT and COPD] were interviewed using a structured questionnaire to assess the consequences of the diseases on household livelihoods, healthcare-seeking behaviours, and coping strategies. A questionnaire was designed to gather information on the consequences of the diseases on the affected individuals and their households, healthcare-seeking behaviors, overall implications for household livelihoods, and coping strategies adopted. Questions were included to assess if the patient unable to do work during the illness. If yes, the interviewee was asked how long she or he was unable to do work during the illness. If the patient faced financial problems due to not being able to work, questions were asked to assess the ADL score and to quantify the financial problems. The questionnaire was pretested before administration. Experienced interviewers administrated the questionaires. They received both classroom and field training on collection of data from hypertension and COPD patients.

### Qualitative techniques

#### In-depth interviews

In-depth interviews to ask about the financial household conquences were conducted among 24 hypertension and 24 COPD patients, stratiefied by gender, income status and age. They were from both study sites. Experienced qualitative researchers were involved in data-collection. Intensive training was imparted to them on data-collection through the techniques mentioned above. They used formal guidelines. The interviewers explained the objectives of study, why the participant was selected, what type of question would be asked during interview with the patients and took consent before starting of interviews. The key area of interest included: assessment of financial assets of households at the time when the illness started and how these changed as the illness progressed, with increased expenditure on healthcare and/or decreased income generation; and an exploration of treatment-seeking patterns, how these changed over time, and what variables affected these.

#### Key-informant interviews

Using information from the sample survey, a purposive sample of most commonly-used providers was drawn to undertake key-informant interviews of their characteristics and advice, treatment, and referral practices in relation to COPD and hypertension. In total, 15 providers of various categories, selected from the community, sub-district, district and tertiary-level facilities from each site, were interviewed. The commonly involved providers were selected based on analysis of survey data. During survey, the patients were asked the name and address of service providers to whom they mostly visit for their treatment. The service providers whose names mostly mentioned by the patients were selected for key informant interviews. It involved a detailed assessment of providers, focusing on their capacity to provide advice, treatment, and referral services in relation to COPD and hypertension. Data on socioeconomic and demographic characteristics of service providers were also collected. They were asked about their training and career history. A semi-structured questionnaire allowed exploration of experience and confidence in treating hypertension or COPD conditions. Referral linkages to the public health system were particularly examined. Information was also collected on the formal service-delivery system and the extent to which the system treats hypertension and COPD conditions.

### Data analyses

Data were entered using the customized visual BASICS/FoxPro software 9.0 and were analyzed using the SPSS software (version 19). The precision of estimates from the quantitative surveys were based on 95% confidence interval (CI). Bivariate and multivariate analyses were carried out, with statistical significance based on 95% CL. Logistic regression analysis was conducted to identify factors that were significantly associated with consequences of the diseases and care-seeking behaviors.

Qualitative information collected through in-depth interviews and key-informant interviews were transcribed, translated into English, and analyzed using contents analysis. Analysis of qualitative data was begun with the first field activities and led to refinements as the study proceeded. From the beginning, we conducted thematic analysis to understand care seeking behaviours and health systems response. The data collector prepared transcript after completion of each interview. The data processing included reading, coding, displaying, reducing, and interpreting. The reading and coding were initiated while data were collected. The primary themes and sub-themes were identified through initial coding. After reading, re-reading, and coding the text, the primary themes and sub-themes were merged with the main themes. When the main themes were formalized, we performed matrix analysis for displaying the data. However, the data-display and reduction process was conducted once all data were collected.

### Ethical clearance

All the respondents gave written consent before participating in the study. The Research Review Committee and the Ethical Review Committee of icddr,b approved the study. The interviews conducted interviews in isolated places and discussed between interviewer and interviewee. Confidentiality of data was strictly maintained.

### Statement of compliance to the RATS guideline for qualitative component

We follow the RATS guideline for qualitative component of the manuscript (please see the RATS checklist provided as an Additional file 1).

## Results

### Sociodemographic characteristics of respondents

Age and sex distribution of clinically-diagnosed patients showed marked variations by type of morbidity and sites (Table 1). The urban patients were younger than the rural patients having each type of morbidity. The patients having hypertension or COPD were younger than the patients having comorbidity in each site. As expected, more males had COPD and comorbidity than females who had more often hypertension in both the sites. In each site, patients with COPD and comorbidity were more likely to have no formal education while patients with hypertension were more likely to have secondary or more education.

**Table 1:** Age, sex, and educational distribution of patients with hypertension, COPD, and comorbidity in urban and rural sites

**Age, sex, and education**	**Dhaka (urban)**			**Matlab (rural)**		
	**COPD**	**Hypertension**	**Comorbidity**	**COPD**	**Hypertension**	**Comorbidity**
**Age**	54.4 ± 15.1	55.5 ± 13.7	63.2 ± 10.2	63.6 ± 13.5	62.3 ± 11.9	68.9 ± 10.8
Mean ± std						
Median	54.6	56.3	62.5	64.4	62.8	69.6
% of female patients	25.0	53.3	32.1	24.7	57.9	32.4
**Level of education**						
% of none	46.2	22.0	35.8	43.5	29.3	41.9
% of primary (class I-V)	25.5	26.0	34.0	37.3	40.7	32.4
% of secondary + (class VI+)	28.2	52.0	30.2	19.2	30.0	25.7
No. of patients (N)	184	246	53	292	140	74

### Consequences of COPD and HT

#### Activities of daily living scores

Patients with COPD and comorbidity has lower ADL scores than those with hypertension in both the urban and rural sites (Table 2). Logistic regression analysis showed higher odds ratio for lower ADL in relation to comorbidity as compared to hypertension in the rural site only. While the age did not show any association with ADL lowering, more males than females had low ADL scores in the rural site (data not shown). The qualitative findings revealed that two-thirds (n = 24) of COPD patients could not perform daily activities, such as bathing, taking food by own hand, wearing dress, performing prayer, cooking, cleaning, gardening, and cattle rearing, and one-fourth had restricted movement.

**Table 2:** **Interruptions in ADL (activities of daily living) of hypertension and COPD patients in rural and urban sites: prevalence (%) and odds ratio (OR) with 95**% **confidence interval (CI) by demographic and socioeconomic variables**

**Type of disease and demographic and socioeconomic variables**	**Dhaka (urban)**		**Matlab (rural)**	
	**ADL interrupted**	**OR**^**1 **^**(95% CI)**	**ADL interrupted**	**OR**^**1 **^**(95% CI)**
**Any patient**	11.2	Not applicable	8.9	Not applicable
COPD	15.2	1.35 (0.69-2.66)	9.6	1.94 (0.78-4.82)
Hypertension (reference)	8.1	1.00	5.0	1.00
Comorbidity	11.3	1.10 (0.40-3.02)	13.5	3.04* (1.07-8.65)
No. of patients (N)	483		506	

^1^The dependent variable was coded as 1 if ADL was interrupted, and coded 0 otherwise; and the odds ratios were adjusted for age, sex, education and asset score of the patients. *p < 0.05.

#### Financial crises

Compared to the rural patients, significantly higher proportion of urban COPD patients reported facing financial crises (12.5% vs. 2.4%, p < 0.01). Of the patients with comorbidity, none reported any household financial crisis in either site. They might be too old to contribute to household economy; other members managed household’s expenditure. The most common option the urban households adopted to cope with economic crises was borrowing money from relatives or friends, followed by reducing expenditure on food and spending or reducing savings (Table 3).

**Table 3:** Prevalence (%) of economic consequences for COPD, hypertension, and comorbidity and coping mechanisms in rural and urban sites

**Economic crises and coping mechanisms**	**Dhaka (urban)**			**Matlab (rural)**		
	**COPD**	**HT**	**Comorbidity**	**COPD**	**HT**	**Comorbidity**
Faced some financial problems	12.5**	3.3	0.0	2.4	1.4	0.0
**Coping mechanism**^**$**^						
Sold household assets	1.1**	0.4	0.0	0.3	0.7	0.0
Spent/reduced savings	4.3**	1.2	0.0	0.0	0.7	0.0
Reduced expenditure on food	6.5**	1.2	0.0	0.7	0.7	0.0
Borrowed money from relative/friend	7.1**	2.0	0.0	1.0	0.0	0.0
Others	0.5	0.4	0.0	0.3	0.0	0.0
No. of patients (N)	184	246	53	292	140	74

^$^Multiple responses were possible; **p < 0.01 (the prevalence compared between sites).

##### Healthcare-seeking behaviours

Seeking medical treatment from any provider in the last one year was higher in the rural than urban patients (Table 4). The quality of medical treatment was associated with urbanity and type of disease. For example, the rate of seeking treatment from medical doctors was higher among the urban patients than among the rural patients, who sought a higher rate from untrained village doctors and drug-sellers. Health workers were seldom consulted for treatment and so were homeopaths, *kabiraj* (practitioner of ayurvedic medicine), and *hekim*s (practitioner of Unani medicine). The patients expressed their satisfactions with the treatment they received from the providers. The number of hospital admissions due to illness in the last one year was few (1-2%) (data not shown).

**Table 4:** Prevalence (%) of seeking medical care for COPD, hypertension, and co-morbidity in last one year by type of provider and level of satisfaction in rural and urban sites

**Type of medical care and level of satisfaction**	**Dhaka (urban)**			**Matlab (rural)**		
	**COPD**	**Hypertens-ion**	**Comorbidity**	**COPD**	**Hypertension**	**Comorbidity**
Did not seek medical care	48.4	48.8	3.8	32.5	41.4	0.0
Sought medical care	51.6**	51.2	96.2	67.5	58.6	100.0
**Type of provider**^**$**^						
MBBS doctor	22.6**	32.5**	18.9	7.9	15.7	9.5
Village doctors/drug stores	25.3**	17.9**	75.5	58.9	41.4	90.5
Health worker	0.0	3.3	1.9	0.3	1.4	0.0
Homeopath/*kabir*aj/*hekim*	1.6	0.0	0.0	0.0	0.0	0.0
# of patients who sought medical care	95	126	51	197	82	74
Satisfied with treatment	93.8**	98.4	100.0	100.0	96.2	100.0
Not satisfied	3.3	0.8	0.0	0.0	2.1	0.0
No. of patients (N)	184	246	53	292	140	74

^$^Multiple responses were possible; **p < 0.01 (the prevalence compared between categories of the variables within the site).

Qualitative data showed that self-treatment at the initial stage of their illness was common among both rural and urban patients with hypertension and COPD. The frightening supernatural force was most often responsible for delaying treatment in Matlab (rural setting). They believe that breathlessness is just a factor of getting older, and the disease does not affect people aged less than 40 years. They sought treatment from any available healthcare provider when the disease became severe. Socially-recognized village doctors in nearby tiny medicine shops were the first option for seeking treatment. The service providers pointed out the patients of either COPD or HT are not under go for treatment. They used to go to service providers whenever they are in severe condition. One service provider with whom indepth interview conducted stated that:

“They (HT patient) don’t understand the consequence of HT. There is lack of awareness among them. So this type of patient come to us suddenly when the condition becomes severe and take medicines. After two or three days whenever they feel better they don’t feel to take any medicine saying that the HT has gone, so when it needs then I will buy the medicine again.”

Patients, particularly uneducated ones, had no clear idea about the technical quality of village doctors. They locally called them *dactar.* One of them recognized a medical representative as an MBBS doctor (formally trained/graduated doctor).

None of the patients with whom in-depth interviews were conducted in rural Matlab knew that treatment of hypertension and COPD could be found from the local public healthcare facilities (subdistrict-level hospitals). A few patients in urban Dhaka knew the names of some tertiary-level hospitals where services for hypertension and COPD are available.

#### Differentials in healthcare seeking

Within the site, consulting trained providers did not vary significantly by type of morbidity, age and education of patients (Table 5). More females than males consulted trained providers. Household asset quintile was the primary determinant of consulting trained providers in both sites; the higher the asset quintile the higher was the consultation rate.

**Table 5:** Medical care sought from trained providers in rural and urban sites: percentage and odds ratio by type of morbidity and socioeconomic and demographic variables

**Type of illness and demographic and socioeconomic variables**	**Dhaka (urban)**		**Matlab (rural)**	
	**Medical care**	**OR**^**1 **^**(95% CI)**	**Medical care**	**OR**^**1 **^**(95% CI)**
Type of patients/illness				
COPD	23.9	0.89 (0.54-1.47)	8.2	0.59 (0.30-1.15)
Hypertension (reference)	32.9	1.00	17.1	1.00
Comorbidity	20.8	0.60 (0.27-1.31)	9.5	0.60 (0.24-1.53)
Age (years) of patients				
35-49	27.0	0.97 (0.54-1.73)	5.9	0.53 (0.15-1.84)
50-59	32.4	1.46 (0.89-2.38)	14.2	1.33 (0.70-2.54)
60+ (reference)	26.0	1.00	10.7	1.00
Sex of patients				
Male (reference)	20.1	1.00	9.7	1.00
Female	40.2	2.30** (1.45-3.66)	13.0	1.20 (0.63-2.29)
Education (class passed)				
None (reference)	28.5	1.00	9.1	1.00
Primary (class I-V)	26.4	1.30 (0.72-2.35)	10.5	1.21 (0.59-2.47)
Secondary + (class VI+)	29.1	0.70 (0.39-1.25)	14.5	1.12 (0.53-2.33)
Household asset quintile				
Lowest (reference)	11.3	1.00	4.0	1.00
Second	20.8	1.83 (0.81-4.16)	4.0	0.93 (0.22-3.92)
Middle	22.1	2.44* (1.01-5.92)	14.7	3.75* (1.14-12.31)
Fourth	38.1	5.91** (2.69-12.96)	13.0	3.46* (1.07-11.20)
Highest	43.8	7.50** (3.26-17.23)	19.5	5.17** (1.54-17.33)
No. of patients (N)	483		506	

^1^The dependent variable was coded as 1 if treatment was sought from a trained provider, and coded 0 otherwise; *p < 0.05; **p < 0.01.

#### Reasons for not seeking treatment

About 49% of the urban patients with COPD and hypertension did not seek any medical care in the last one year, and it was less (32% and 41% respecticely) in the rural patients. The single most common reason they stated was lack of need for seeking care (>85%), followed by lack of money, 13% for COPD in the urban site (data not shown).

#### Household service expenditures

The average out-of-pocket expenditures per visit were higher for urban than rural patients for COPD and HT and opposite for co-morbidity (Table 6). The expenditures were higher for female than male patients in the urban site and the male–female difference was small in the rural site. Treatment expenditure that includes consultation fee and costs for diagnostic test and medicines.

**Table 6:** **Out-of-pocket expenditures (in taka**^**a**^**) per visit for seeking non-domiciliary treatment for HT, COPD and co-morbidity by area**

**Type of illness and sex of the patients**	**Dhaka (Urban)**				**Matlab (Rural)**			
	**Overall**		**% of overall mean**		**Overall**		**% of overall mean**	
	**N**	**mean**	**Treatment**^**b**^	**Travel**	**N**	**mean**	**Treatment**^**b**^	**Travel**
Any illness	463	1749	96.8	3.2	686	371	92.2	7.8
HT	202	1385	94.4	5.6	132	525	83.8	16.2
COPD	153	3275	98.3	1.7	377	342	96.2	3.8
Co-morbidity	108	269	94.4	5.6	177	319	93.4	6.6
Sex of the patients								
Male	239	1054	95.6	4.4	432	367	94.0	6.0
Female	224	2491	97.4	2.6	254	378	89.1	10.8

N = number of visits, ^a^Taka 78 = US$1, ^b^treatment cost includes consultation fee and costs of diagnostic test and medicines.

### Health system responses

#### Service facilities

##### Pharmacy/medicine shops

Although some pharmacies/medicine shops possessed machines for checking blood pressure with a fee, they did not have any equipment to diagnose COPD.

##### Government hospitals

The government upazila (sub-district) hospital in Matlab has facilities for performing basic pathology tests, such as urine, stool, and blood, doing x-rays, and machines for measuring blood pressure; however, it had no equipment for the diagnosis of COPD. The Union Health and Family Welfare Centres are equipped with blood pressure-measuring machines but these centres had no instrument to diagnose COPD. The providers offer treatment to COPD patients based on symptoms, often considering these as asthma.

##### Private clinics

The MBBS doctors of private clinics usually concentrated on follow-up of hypertension patients. They are used to ask patients with hypertension to visit them for follow-up every month. According to them, only measuring blood pressure routinely every month is enough as a routine service for hypertension patients. They neither had any equipment nor any training to manage COPD patients.

##### Specialized hospitals

The doctors of specialized hospitals mainly expressed their intensions to find out the cause of hypertension in patients. After proper diagnosis and obtaining the patient’s history, they tried to identify the cause(s). To get the outdoor services, patients need to buy a ticket by paying Tk 10 (US$ 0.07). If the attending doctor advices any patients for any laboratory test, they need to do this from the outside diagnostic centres and could meet the doctor again as per the need.

#### Provider training

None, except the service providers, at the specialized hospitals had any training on the diagnosis and management of patients with hypertension and COPD. The service providers of primary healthcare facilities provide services to patients based on their basic training they received at the time of their joining the service. Hypertension and COPD are currently being common conditions among many patients, the government MBBS doctors of all tiers of the health system emphasize on training on the management of hypertension and diagnoses and management of COPD. They especially recommended training on type and dose of drug for the treatment of hypertension to know about the appropriate latest medicines for managing a specific condition of patients to reduce their misuse. Despite having better knowledge, other service providers in both government and private clinics asked for the latest treatment of hypertension and COPD. They added that training of government field workers currently not involved in any activity to prevent and control chronic diseases is required so that they can motivate people about the prevention and treatment of hypertension and COPD.

The village doctors/drug-sellers at pharmacies also urged for training on screening, treatment, and referral of hypertension and COPD patients. They added that, since they stay in the community, they are the first contact points for patients at the initial stage. They should have, thus, good knowledge about the diseases so that they can motivate patients, advice them about prevention and provide treatment.

#### Referral system

In urban Dhaka, government MBBS doctors, practicing in private chambers, did not refer any hypertensive patient to any other health facility, since they have the facility to manage patients even with cardiac problems. They reported that the local people lack knowledge as to which type of doctors or hospitals they should visit for a specific disease. So, being a doctor they referred hypertension patients diagnosed with any kidney-related problem to the kidney disease hospital. As private practitioners practice in private clinics, they refer their hypertension patients only when the disease condition is severe or deteriorates.

Pharmacy vendors generally recommended serious patients to visit the Dhaka Medical Collage Hospital (DMCH) or the Shaheed Suhrawardy Medical College (SSMC). One of them stated:

“Most pharmacy-based practitioners did not want to take any risk for any serious patient. They referred them to the two hospitals (DMCH or SSMC). So, without wasting the patients’ time, we directly send them to those hospitals”.

They generally recommended visiting a nearby private hospital. However, they referred poor patents to the DMCH because it was not cost-effective for patients to be treated in a private hospital. The MBBS doctors who practice in private clinics mentioned that there is no need to refer any hypertension patient to any other facility.

#### Follow-up systems

Generally, in the absence of any mechanism, government or private doctors cannot confirm whether the referred patient visited the referred facility or not. Pharmacy vendors were in a better position to know about the status of some patients if they came again to them to buy medicines with prescriptions. On the contrary, one of the government doctors questioned about the importance of knowing the patient’s referral status. According to him, it might be the responsibility of the patient, otherwise the patient will suffer.

#### Provider suggestions for treatments

The healthcare providers recommended the following actions or steps to be taken for the treatment of hypertension and COPD:

• To make available and improve the services for chronic diseases at the public-sector primary healthcare facilities, especially in rural sites, are essential to ensure services for the disadvantaged population of Bangladesh.

• To supply an adequate number of medical equipment for the diagnosis of chronic diseases in the health facilities must be ensured. Nebulization support needs to be made available at the outdoor facilities for providing emergency services to patients. Supply of other medicines should be ensured for the treatment of chronic diseases at every healthcare facility both at public and private hospitals.

• To make available ECG and X-ray machines and technicians at district- and subdistrict-level hospitals should be ensured as these are life-saving instruments. Any emergency heart or COPD patient cannot be referred to any other facility without proper checking and diagnosis.

• To increase the reliability on hospital services, a corner inside primary healthcare-level hospitals should be developed for patients with COPD, hypertension, and other chronic diseases. Besides, it needs media exposure so that people are aware of it and visit it for services instead of seeking services from village doctors and pharmacies.

• To increase awareness among the community people about hypertension and COPD and their prevention and treatment are important. The involvement of community health workers in the prevention and control of hypertension and COPD patients is essential. However, currently there is no program to involve community health workers to prevent and control chronic diseases.

• To increase pathology services which can be performed without electricity at union subcenters need to be arranged.

• To provide free treatment of hypertension and COPD patients or providing medicines at reduced prices, like through the directly-observed therapy of tuberculosis (DOTs) programme should be introduced to control chronic diseases.

• To develop a risk-reduction strategy for COPD patients in the anti-tobacco campaigns.

## Discussion

Our study reports the important health and social consequences of HT and COPD, the health care seeking behaviour of patients and responses of health providers in Bangladesh.

We found lower ADL scores among patients of COPD than among those with hypertension and their combined effects. Results of logistic regression showed significantly higher OR of low ADL scores in case of comorbidities as compared to those with hypertension.

Qualitative information shows more ADL impairment among the patients than the quantitative ADL scores. This might be due to more probing during qualitative data collection. Two-thirds of the COPD patients reported their inability to perform daily activities in various forms, such as bathing, taking food by own hand, getting dress, and praying. Many reported that COPD restricted their movements. Due to this physical condition, most of them could not carry out their daily household activities, such as cooking, cleaning, gardening, and cattle rearing.

There were high out-of-pocket expenditure and loss of income. Self-treatment of conditions during the initial stage was common in all the groups. Our study showed that the chronic conditions warranted high out-of-pocket expenditure and loss of wage or income more in the urban site than the rural site. The most common option households adopted to cope was borrowing money from relatives or friends, followed by reducing expenditure on food and reducing savings.

Around half of both rural and urban cases did not seek any medical treatment for COPD and HT. Many did not realize that they had the condition, mistakenly believing that breathlessness is just a factor of getting older, and the disease does not affect people aged less than 40 years. Many believed they were merely suffering from ‘smoker’s cough’, which is not important enough to bother their doctors. These findings are also consistent with findings of a study by the World Health Organization [18]. The most common reason for not seeking any treatment was lack of felt-need for seeking care, followed by lack of money. A large majority sought treatment from pharmacies and from untrained village doctors. Seeking services from public-sector doctors was rare in rural Matlab and very few in urban Dhaka. Lack of awareness, information, education, and motivation might have played a role for not seeking treatment. The primary resorts for communities were typically pharmacies/drugstores and a range of informal providers from traditional healers to semi- or untrained village doctors or’quacks’. We observed a limited access of qualified biomedical providers by the poor. The key factors that affect health-seeking behaviors include knowledge about conditions and available services in the locality, cost of treatment and availability of finance, cultural preferences, and socioeconomic and gender relationships. Rigorous awareness raising programmes involving community health workers both in rural and urban areas may contribute in prevention and control HT and COPD.

Seeking care from formally-trained providers was higher in an urban setting and was higher for hypertension. The referral mechanism for COPD and hypertension patients was inadequate, and many were not referred until the severity of diseases increased. The qualitative data showed that unidentified COPD placed a substantial burden on patients, resulting in decreased lung function and associated symptoms, and led to physical impairment.

Our findings are consistent with findings of a study by Ahsan Karar in Bangladesh [16]. It is clear from above findings that COPD and HT have impacts on ADL and financial situation of patients, make the patients vulnerable in terms of physically and economically. Therefore, policy level should take immediate steps to develop programmes on prevention and control of COPD and HT.

The limitation of the study was that the sample of qualitative component were selected purposively and there may be a risk of selection bias. However, for minimizing the selection bias, sampling was done considering geographical areas (rural and urban), age (young adult and old) and gender (males and females).

The important factors relating to responses of providers included background and (both formal and non-formal) training, position in the local’market’ for services, relationships with members of the local community and relevant authorities, degree of specialization, and links to formal service provision. Except the specialized hospitals, the healthcare facilities are not well-equipped; the providers had no adequate training on the diagnosis and management of hypertension and COPD patients; and there was no referral and follow-up system for patients. These need special attention of the MoHFW of the Government of Bangladesh. The MoHFW should ensure treatment for HT and COPD from trained providers. Further, healthcare facilities at PHC level needs to equipt to responed the needs of patients with COPD and HT.

## Conclusions and implications

Few studies have explored the health consequences of HT and COPD, care seeking behaviour of patients, and the health system responses in Bangladesh, not in other low-income settings. In Bangladesh, no standard and evidence-based program is available for providing services to patients with hypertension and COPD, except in some very limited specialized hospitals at the tertiary levels. The findings of our study revealed that at PHC level, the health systems of Bangladesh are not yet ready to respond to the needs of patients with hypertension and COPD. On the other hand, although MoHFW has recently taken some initiatives, there is no strategic approach to include these diseases in the primary healthcare structure. However, PHC facilities should be equped to address the needs of HT and COPD patients and to reduce household level poverty. Active involvement of community health workers in prevention and control of HT and COPD may be a good strategy. Research to assess the effectiveness of such strategies is essential before proceeding to their large-scale implementation.

## Competing interests

The authors declare that they have no competing interests.

## Authors’ contributions

JU, LN, NA, and DSA were involved in developing the concept and implementation of the study, analysis of data, and writing of the manuscript. HS was involved in analysis of qualitative data and writing of the manuscript. JU and AHC were also involved in the collection and processing of data. All the authors read and approved the final manuscript.

## Pre-publication history

The pre-publication history for this paper can be accessed here:

http://www.biomedcentral.com/1471-2458/14/547/prepub

## Supplementary Material

Additional file 1 Qualitative research review guidelines – RATS. Click here for file

## References

[B1] World Health Organization Global Status Report On Non-Communicable Diseases 2010 2011 Geneva: WHO Press 1 11

[B2] World Health Organization World Health Statistics 2012. Part II, Highlighted Topics 2012 Geneva: WHO Press 34

[B3] Parfitt B Health reform, the human resource challenges for central Asian Commonwealth of independence States (CIS) Countries Collegian 2009 16 35 40 10.1016/j.colegn.2009.01.002 19388425

[B4] Vestbo J Hurd SS Agusti AG Jones PW Vogelmeier C Anzueto A Rodriguez-Roisin R Global strategy for the diagnosis, management, and prevention of chronic obstructive pulmonary disease: GOLD executive summary Am J Respir Crit Care Med 2013 18 4 347 365 22878278 10.1164/rccm.201204-0596PP

[B5] Lawes CM Hoorn SV Rodgers A Global burden of blood-pressure-related disease, 2001 Lancet 2008 371 9623 1513 1518 10.1016/S0140-6736(08)60655-8 18456100

[B6] icddr,b Chronic disease news: a news letter of icddr,b 2013 5 1 1 6 [http://www.icddrb.org/what-we-do/publications/cat_view/52-publications/10042-icddrb-periodicals/10079-chronic-disease-news/14388-vol-5-issue-1-english-2013?orderby=dmdate_published&ascdesc=DESC]

[B7] Hossain SM Boonshuyar C Ekram ARMS Non-adherence to antihypertensive treatment in essential hypertensive patients in Rajshahi, Bangladesh AKMMC J 2011 2 1 09 14

[B8] Abegunde D Maters C Adam T Ortegon M Strong K The burden and costs of chronic diseases in low-income and middle-income countries Lancet 2007 370 1929 38 10.1016/S0140-6736(07)61696-1 18063029

[B9] Khanam MA Lindeboom W Koehlmoos TP Hypertension: Adherence To Treatment In Rural Bangladesh--Findings From A Population-Based Study (Abstract) Abstracts Book of the 13th ASCON 2011 Dhaka: icddr,b 18 10.3402/gha.v7.25028 PMC4212079 25361723

[B10] Mannino DM Buist AS Global burden of COPD: risk factors, prevalence, and future trends Lancet 2007 370 9589 765 73 10.1016/S0140-6736(07)61380-4 17765526

[B11] World Health Organization Fact Sheet 2012

[B12] Bangladesh. Ministry of Health and Family Welfare. Directorate General of Health Services Strategic Plan For Surveillance And Prevention Of Non-Communicable Diseases in Bangladesh 2007–2010 2007 Dhaka: Directorate General of Health Services, Ministry of Health and Family Welfare, Government of Bangladesh 1 5

[B13] Sayeed MA Banu A Haq JA Khanam PA Mahtab H Azad Khan AK Prevalence of hypertension in Bangladesh: effect of socioeconomic risk factor on difference between rural and urban community Bangladesh Med Res Counc 2002 28 1 7 18 12587756

[B14] Parr JD Witzelindeboom Khanam MA Koehlmoos TP Diagnosis of chronic conditions with modifiable lifestyle risk factors in selected urban and rural areas of Bangladesh and sociodemographic variability therein BMC Health Serv Res 2011 11 309 10.1186/1472-6963-11-309 22078128 PMC3239323

[B15] World Health Organization Non-Communicable Disease Risk Factor Survey Bangladesh 2010 2011 Bangladesh: WHO Press 1 24

[B16] Ahsan Karar Z Alam N Streatfield PK Epidemiological Transition in Rural Bangladesh, 1986–2006 2009 Global Health Action: Dhaka 10.3402/gha.v2i0.1904 PMC2779938 20027273

[B17] Swedish International Development Cooperation Agency Reality Check Bangladesh 2009: Listening To The Poor People’s Realities About Primary Healthcare And Primary Education 2010 Dhaka: Edita Communications

[B18] World Health Organization. Department of Public Health and Environment Indoor Air Pollution: National Burden Of Disease Estimates 2007 Geneva: World Health Organization

