# Erratum to: Consequences of hypertension and chronic obstructive pulmonary disease, healthcare-seeking behaviors of patients, and responses of the health system: a population-based cross-sectional study in Bangladesh

**DOI:** 10.1186/1471-2458-14-823

**Published:** 2014-08-09

**Authors:** Md Jasim Uddin, Nurul Alam, Tracey P Koehlmoos, Haribondhu Sarma, Muhammad Ashique Haider Chowdhury, Dewan S Alam, Louis Niessen

**Affiliations:** International Centre for Diarrhoeal Disease Research Bangladesh (icddr,b), Mohakhali, Dhaka, 1212 Bangladesh; Headquarter Marine Corps, Pentagon, Washington, DC 20530-3000 USA; Centre for Applied Health Research and Delivery, Suite 1966–206, Liverpool School of Tropical Medicine/Medical School, University of Warwick Pembroke Place, Liverpool, L3 5QA UK

## Correction

The authors would like to apologise for not including Tracey P. Koehlmoos as an author on this work [1]. She was involved in the conception and design of the study and should have been included in the author list. This has now been corrected and the author list, authors’ contribution and competing interests sections have been updated as below:
