# Supplementary material for: Consequences of hypertension and chronic obstructive pulmonary disease, healthcare-seeking behaviors of patients, and responses of the health system: a population-based cross-sectional study in Bangladesh
Source: BMC Public Health. 2014 Jun 3;14:547. doi: 10.1186/1471-2458-14-547 (PMC4049371; doi:10.1186/1471-2458-14-547)
Supplement: Additional file 1 — Qualitative research review guidelines – RATS. [file 1471-2458-14-547-S1.docx]

**Qualitative research review guidelines – RATS**

| **ASK THIS OF THE MANUSCRIPT** | **THIS SHOULD BE INCLUDED IN THE MANUSCRIPT** |  |
| --- | --- | --- |
| **R Relevance of study question** |  |  |
| Is the research question interesting?  Is the research question relevant to clinical practice, public health, or policy? | Research question explicitly stated  Research question justified and linked to the existing knowledge base ( policy) |  |
| **A Appropriateness of qualitative method** |  |  |
| Is qualitative methodology the best approach for the study aims?   - *Interviews:* experience, perceptions, behaviour, practice, process - *Focus groups:* group dynamics, convenience, non-sensitive topics - *Ethnography:* culture, organizational behaviour, interaction - *Textual analysis:* documents, art, representations, conversations | Study design described and justified i.e., why was the particular method (e.g., interviews) chosen |  |
| **T Transparency of procedures**  *Sampling* |  |  |
| Are the participants selected the most appropriate to provide access to the type of knowledge sought by the study?  Is the sampling strategy appropriate? | Criteria for selecting the study sample justified and explained   - *purposive:* diversity of opinion - *volunteer:* feasibility, hard-to-reach groups |  |
| *Recruitment* |  |  |
| Was recruitment conducted using appropriate methods? | Details of how recruitment was conducted and by whom are provided |  |
| Is the sampling strategy appropriate? | Yes |  |
| Could there be selection bias? | Details are written |  |
| *Data collection* |  |  |
| Was collection of data systematic and comprehensive? | Method(s) outlined and examples given (e.g., interview questions) clearly described |  |
| Are characteristics of the study group and setting clear? | Study group and setting clearly described |  |
| Why and when was data collection stopped, and is this reasonable? | End of data collection justified and described |  |
| *Role of researchers* |  |  |
| Is the researcher(s) appropriate? How might they bias (good and bad) the conduct of the study and results? | Do the researchers occupy dual roles (clinician and researcher)? Are the ethics of this discussed? Do the researcher(s) critically examine their own influence on the formulation of the research question, data collection, and interpretation?  Provided detail description |  |
| *Ethics* |  |  |
| Was informed consent sought and granted? | Informed consent process explicitly and clearly detailed |  |
| Were participants’ anonymity and confidentiality ensured? | Anonymity and confidentiality discussed |  |
| Was approval from an appropriate ethics committee received? | Ethics approval cited |  |
| **S Soundness of interpretive approach**  *Analysis* |  |  |
| Is the type of analysis appropriate for the type of study?   - *thematic:* exploratory, descriptive, hypothesis generating - *framework:* e.g., policy - *constant comparison/grounded theory:* theory generating, analytical   Are the interpretations clearly presented and adequately supported by the evidence? | Analytic approach described in depth and justified  *Indicators of quality:* Description of how themes were derived from the data (inductive or deductive)  Evidence of alternative explanations being sought  Analysis and presentation of negative or deviant cases |  |
| Are quotes used and are these appropriate and effective? | Description of the basis on which quotes were chosen  Semi-quantification when appropriate  Illumination of context and/or meaning, richly detailed |  |
| Was trustworthiness/reliability of the data and interpretations checked? | Method of reliability check described and justified e.g., was an audit trail, triangulation, or member checking employed? Did an independent analyst review data and contest themes? How were disagreements resolved? |  |
| *Discussion and presentation* |  |  |
| Are findings sufficiently grounded in a theoretical or conceptual framework?  Is adequate account taken of previous knowledge and how the findings add? | Findings presented with reference to existing theoretical and empirical literature, and how they contribute |  |
| Are the limitations thoughtfully considered? | Strengths and limitations explicitly described and discussed |  |
| Is the manuscript well written and accessible? | Written for a health sciences audience |  |
|  |  |  |
|  |  | |
